# A Difficult and Complicated Obstetrical Case

**Published:** 1887-03

**Authors:** R. H. Eanes

**Affiliations:** Rice’s Crossing, Texas


### A DIFFICULT AND COMPLICATED OBSTETRIC CASE.

*By R. H. Eanes, M. D., Rice's Crossing, Texas.*

For Daniel's Texas Medical Journal.

ON the morning of 2nd inst. I was called up about 4 o'clock by a Bohemian who, to use his own language, said: "Wife much sick; child no come." I understood him at once and hastened to his house, knowing too well that physicians were not called by them in cases of labor unless some great trouble or imminent danger existed. I was met at the door by a Bohemian mid-wife, who informed me in broken English that the water had long since come away, but the child would not advance. I made an immediate examination and discovered a footling presentation with a prolapsed cord. Upon further examination I made out a sacro-posterior position, but whether right or left I do not pretend to say.

The left foot was protruding and the right one just within the vulva. There were twelve or eighteen inches of prolapsed cord, large, engorged and convoluted. Its size and shape reminded me at once of the small intestine. My first effort, before attempting delivery, was to replace the cord. In this I failed, for as fast as I could replace a knuckle another one would slip down. Its enormous size and weight rendered all attempts in this direction perfectly hopeless. At this time the cord was beating feebly. One marked peculiarity of the case was the entire absence of uterine pains. This, with the failure to replace the cord, puzzled me as to how I should next proceed. I reasoned thus: the woman has lain all night in labor and all womb power has been exhausted; as a consequence some interference on the part of the accoucheur is now indicated. I gave a good dose ot ergot and awaited developments, occasionally kneading the womb. But there were no developments. All parties present being uneasy and anxious that something should be done, I proceeded to deliver. (I will say just here that I could get no history of the case, as no one present spoke intelligible English). The left foot protruding, I introduced my hand and pulled down the right on a level with the left; with my right hand I now grasped both feet, at the same time using traction; and with my left, exerted strong pressure from above. At this juncture a good pain came on, and the child was born to the navel. Time, now, with the child, I knew to be everything. I waited a few moments for a recurrence of the pain, but when it did not recur I again assisted, using some traction and a good deal of pressure. The womb, however, did not lend her aid, and my efforts availed nothing. I now examined the cord and found its beat so faint as hardly to be perceptible. I again endeavored to deliver, but the womb was inert and the child as fast as if in a workman's vise. The cord was now pulseless, and I almost despaired; but again tried, hoping to be able to resuscitate the child, could I deliver without further delay. Efforts, however, were fruitless, and the woman complained so much of my manipulations that I ceased all efforts. Knowing now that the child was dead, like Micawber, I waited for something "to turn up," but as nothing turned up, I administered chloroform, introduced my hand, swept the arms over the face, (which, by the way, were extended above the head) and then, with renewed traction and pressure, the shoulders came away. But here again things stopped, and the nead was as fast now as the shoulders were before. The truth was, the chin was extended and fixed behind the symphysis. I again introduced my hand and fastening, as well as I could, my index and middle fingers upon the forehead of the child, caused partial rotation backward. By this means I was enabled to get my index finger in the mouth, thus making the rotation complete and the child was born. Of course this gave quite a twist to the neck, but as the child had been dead possibly half an hour no harm could result. The child was of average size, with no deformities. The after-birth came away without trouble, and the womb contracted well.

I presume, Mr. Editor, to report this case, because it is one of the most remarkable with which I have ever had to deal in practice. The footling presentation, the prolapsed cord, the non-rotation forward of the occiput, and the fixedness of the chin behind the pubis, made it a most difficult and complicated case, indeed, to deal with. During my short professional career, I have had four cases of breech presentation, but none to resemble this. I would be pleased to have you call the attention of your many readers to the following interesting points:

1. Playfair says this may be done in two ways. The body of the "child being grasped in the intervals of pains and twisted round so as to bring the occiput forward. It is by no means certain, however, that the head would follow the movements imparted to the body, and there must be a serious danger of giving a fatal twist of the neck by such a manœuvre." The other plan, recommended by himself, is the one practiced in this case. It seems to me that if either of these methods alone is good, that a judicious combination of both would produce the result desired. This is the method I would recommend, and would myself practice in a similar case.

2. On due reflection the question has arisen in my mind whether labor in this case had actually begun or not? The feet protruding, the waters having come away, and the "parts" being well dilated, indicated at once, seemingly, that it had. But when we remember the entire absence of pains (with one exception) and that the feet had been advancing and receeding for about fourteen days, actually protruding as they were on the night of delivery. This, (I was informed by an interpretor, after all was over, and by-the-way a very remarkable feature of the case) makes it a question with me. Might not the waters have been emptied by some movement of the child's feet, protruding as they were, or some jostle or injury to the mother, the parts dilated by the gradual insinuation of the feet for those fourteen days? My solution of the question is this: Labor had begun, but was in its incipiency, and like most breech presentations, was necessarilly slow, but without professional interferance would have been born on the evening or the night of the next day.

Granting this, would not the child with the pendulous and feebly beating cord, have become asphyxiated before that time, and was it not more scientific, after all, to interfere?

I should be glad to have the comments of any professional brother on this case.

